# PV resource evaluation based on Xception and VGG19 two-layer network algorithm

**DOI:** 10.1016/j.heliyon.2023.e21450

**Published:** 2023-10-31

**Authors:** Lifeng Li, Zaimin Yang, Xiongping Yang, Jiaming Li, Qianyufan Zhou

**Affiliations:** a Energy Development Research Institute, China Southern Power Grid, China; b China Southern Power Grid, China; c Guangdong Key Laboratory of Green Energy Technology, South China University of Technology, China

**Keywords:** Photovoltaic resource assessment, Convolutional neural networks, Double layer network algorithm, Two-tier network framework

## Abstract

With the increasing global demand for new energy sources, Photovoltaic (PV) is increasingly emphasized as a renewable energy source globally. Consequently, the assessment of PV resources has become crucial. Existing single frameworks and algorithms for PV resource assessment lead to low assessment accuracy. To alleviate the deficiency, this study proposes a two-layer network algorithm based on Xception and VGG19 for the evaluation of PV resources. The proposed method combines Xception convolutional neural network and VGG19 convolutional neural network. In addition, this study constructs a two-layer network framework based on the two-layer network algorithm. The feasibility and reliability of the proposed method are verified by simulating the proposed method under the case. Compared with existing algorithms, the proposed method and framework can improve the accuracy of PV resource assessment.

## 1 Introduction

With the rapid development of new energy sources and the global commitment to the carbon-neutral goal of reaching peak carbon, solar energy as a clean, renewable energy source has the potential for a wide range of applications [1]. To achieve the goal of carbon neutrality, China is vigorously developing new energy sources, of which photovoltaic power generation is an important part [2]. PV resource assessment is a key task, and the accuracy and reliability of PV resource assessment will directly affect the construction and operation of PV power plants, which in turn will affect the achievement of China's peak carbon neutrality target [3]. Accurate and efficient assessment methods will provide strong support for the sustainable development of the PV industry, thereby promoting China's goal of green and low-carbon development [4].

Traditional PV resource assessment methods are vulnerable to data quality and reliability and cannot cope with problems such as complex terrain and weather conditions, resulting in inaccurate and unreliable assessment results, thus affecting the construction and operational benefits of PV plants [5]. Therefore, more advanced methods need to be developed to solve these problems. In recent years, with the development of artificial intelligence and big data technology, methods such as machine learning have been more and more widely used in PV resource assessment [6]. Unlike traditional assessment methods, artificial intelligence can automatically learn the laws and relationships between data and build more accurate prediction models by training a large amount of data [7], and then realize the assessment of PV resources.

Among the existing studies to evaluate PV resources, artificial intelligence-based methods for PV resource evaluation include recurrent neural networks (RNNs) [8], support vector machines (SVM) [9], genetic algorithms (GA) [10], etc. These algorithms may have some problems when evaluating PV resources as follows: (a) High computational complexity: RNNs need to compute the output of each time step, and the computation of each time step depends on the computation results of the previous time steps, so they are computationally intensive and have high hardware requirements [11]; (2) Optimal solution problem: GA requires modeling of the problem and parameter selection, which may result in finding an optimal solution that is not the actual optimal solution if it is not modeled properly or if the parameters are not selected appropriately [12]; (3) Dependence on large amounts of labeled data: machine learning-based algorithms usually require large amounts of labeled data to train models, but for PV resource assessment, labeled data are difficult to obtain or require significant time and effort to obtain [13]; (4) Limited by the limitations of the algorithm itself: a single algorithm may have some limitations, such as being sensitive to specific types of data or noise [14]. These limitations may lead to compromised accuracy and reliability of the model; (5) Lack of data diversity: since the assessment of PV resources needs to consider several factors, including meteorological, geographical, and environmental factors [15], a single algorithm may not be able to cover all data characteristics, which leads to inaccurate assessment results.

In this paper, we propose a PV resource evaluation method based on Xception and VGG19 two-layer network algorithm. The proposed method combines the Xception convolutional neural network [16] and VGG19 convolutional neural network [17] to improve the accuracy and reliability of PV resource assessment by using a two-layer network structure combining the advantages of both Xception and VGG19 algorithms. Compared to a single network structure formed by a single algorithm, the use of a two-layer network can improve the robustness of the model [18] and can better adapt to different data distributions and scenarios. However, the single network structure approach can only capture simple linear or nonlinear relationships [19] and cannot capture more complex relationships, causing the problem of poor robustness of the single network structure approach; due to the complexity and diversity of PV resource assessment data, the single network structure may not be able to effectively capture nonlinear relationships and patterns among the data, leading to overfitting or underfitting problems [20]. In contrast, a two-tier network structure can reduce the risk of overfitting by cross-validating in two different algorithms, thus improving the generalization ability of the model [21].

The main contributions of this paper can be summarized as follows.

(1) The existing frameworks for PV resource assessment are usually single-layer network structure frameworks. However, the two-layer network framework is proposed in this study to replace the two-layer network framework. The proposed two-layer network framework can capture the correlation between PV data features more accurately and possesses a more precise prediction capability. To the best knowledge of the authors, the proposed two-layer network framework is the first novel method found in this study.
(2) A two-layer network algorithm is proposed to replace the existing single network algorithm. The proposed two-layer network algorithm fills the gap of applying only a single algorithm to solve the PV resource assessment.
(3) The two-layer network algorithm can accurately learn the correlation between the input data because of its ability to combine the features with multiple extractions. Compared to the existing PV prediction methods, the proposed method in this study can provide more accurate prediction capability.

The rest of this study consists of the following three parts. The second part introduces the proposed two-layer network algorithm and two-layer network framework in this study; the third part verifies the feasibility and evaluates the performance of the proposed framework and algorithm through simulation examples; the fourth part concludes this study.

## 2 Related works

Xception which is a kind of deep convolutional neural network has made remarkable achievements in its application in the field of engineering. By inputting a large amount of meteorological data, geographic information, and wind turbine performance parameters into the Xception network, it is possible to predict the amount of wind power generation at a certain time in the future, which allows for better scheduling of power resources and improves the stability of the power grid [22]. Besides, the Xception network excels in power equipment troubleshooting. By processing and analyzing image data from power equipment, Xception can detect defects, damage, or wear on the surface of the equipment [23]. In addition, Xception is also utilized for energy load forecasting, where Xception analyzes historical energy usage data, weather information, and building characteristics to provide cities and buildings with optimal energy management strategies to reduce energy waste and lower carbon emissions [24]. Additionally, Xception analyzes sensor data and images of power equipment to enable intelligent maintenance schedules for equipment, extending the life of the equipment, reducing maintenance downtime, and increasing the availability of the power system [25]. Furthermore, Xception improves the reliability of power systems and reduces the likelihood of accidents by analyzing images of power lines and transmission towers to detect possible faults or damage [26].

The VGG19 convolutional neural network has been widely applied in the field of power system engineering, and its powerful performance in image processing and recognition provides important support for tasks such as PV resource assessment. With excellent feature extraction capabilities, VGG19 allows accurate prediction of future solar energy generation by using historical meteorological data and solar radiation data, helping to optimize the operation of solar power stations [27]. Besides, VGG19 with excellent generalization capability improves fault feature recognition by analyzing fault feature maps of grid equipment [28]. In addition, VGG19 improves equipment availability by analyzing equipment sensor data and images to detect abnormal behavior, predict equipment life, and reduce the risk of sudden failures [29]. Additionally, VGG19 analyzes building energy consumption data, indoor environmental conditions, and energy management strategies to provide buildings with optimized energy consumption scenarios that reduce energy costs while reducing environmental impact [30]. Furthermore, VGG19 performs feature extraction on images of historical stock price data trips to predict stock prices.

To utilize PV power generation on a large scale, the prediction of light intensity is particularly important. The intensity of solar radiation is a major influence on the amount of PV power generated. By combining the data from the historical records of radiation intensity and based on the historical weather conditions and other information, the prediction of radiation intensity has been carried out using the method of a single neural network, which has a satisfactory prediction accuracy [31]. In addition, based on emerging PV prediction methods (i.e., satellite images, cloud morphology, and remote sensing techniques) were applied [32]. takes the calculated radiation intensity received by the earth as input and the solar radiation intensity received by the PV panels as actual output. Finally, the prediction of PV power generation is carried out based on the prediction model [33]. In terms of existing prediction methods, on the one hand, a single neural network method cannot obtain more accurate prediction results, and on the other hand, emerging prediction methods need to be utilized in conjunction with emerging technologies and still require strong algorithmic theory. Therefore, a prediction method with high prediction accuracy and simple algorithm theory is needed.

## 3 A PV resource assessment method based on Xception and VGG19 two-layer network algorithm

In the introduction of Part I, this study has introduced the importance of PV resource assessment and its application prospects in the field of renewable energy. To effectively assess the potential and efficiency of PV resources, this study proposes a PV resource assessment method based on the Xception network and VGG19 two-layer network algorithm. In this section, we will introduce the principle and application of this method in detail, to provide a reliable assessment method for the planning and optimization of PV energy systems.

### 3.1 Methods

In this section, we will introduce the Xception algorithm and the VGG19 algorithm. By introducing the principles of the Xception algorithm and the VGG19 algorithm, we will provide the reader with the necessary background knowledge to better understand our proposed PV resource evaluation method based on the Xception and VGG19 bilayer network algorithms.

#### 3.1.1 Xception neural network

**Fig. 1:**
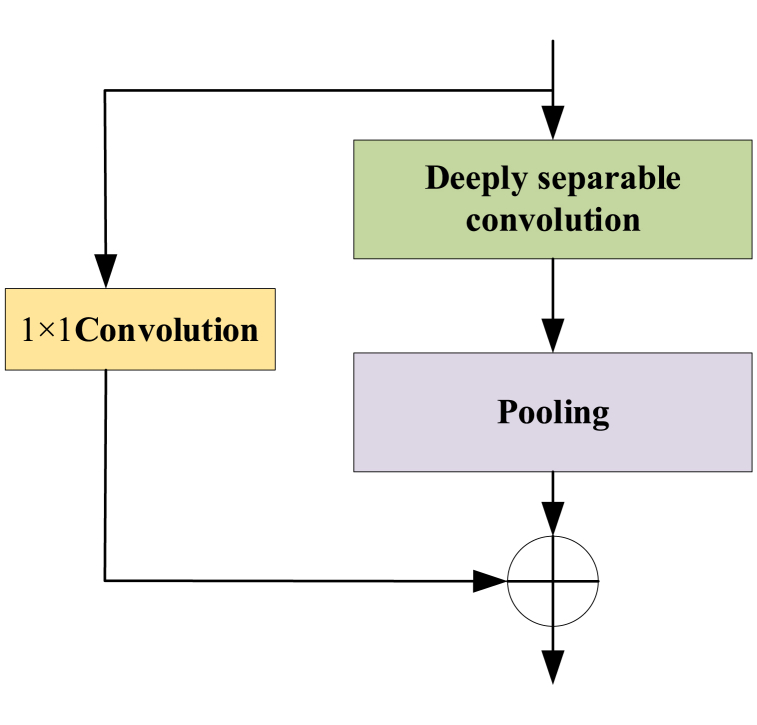
Simplified Xception module.

Xception is a deep convolutional neural network whose main feature is the use of deep separable convolution instead of traditional convolutional operations, thus achieving efficient feature extraction and computation while reducing the number of parameters. The Xception convolutional neural network consists of separable convolutional layers and standard convolutional layers. In this case, deep convolution is used to process information between channels, while point-by-point convolution is used to process information for each channel [34]. This separation allows the network to learn more fully the features between and within the different channels. Deeply separable convolution has been widely used in Xception networks because of its efficient feature extraction capability and low computational complexity. The main structure of the Xception neural network is shown in Fig. 1.

#### 3.1.2 VGG19

**Fig. 2:**
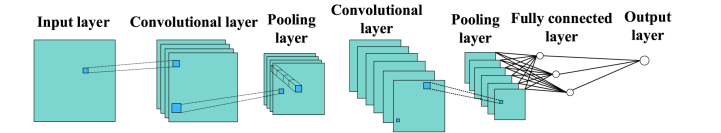
VGG19 structure diagram.

The VGG19 is a deep convolutional neural network whose structure is shown in Fig. 2. The VGG19 consists of a total of 19 convolutional layers and fully connected layers. VGG19 uses multiple convolutional layers [35] and pooling layers [36] in an alternating manner, and uses a small convolutional kernel design to reduce the number of parameters while increasing the depth of the network, which can improve the accuracy of the network.

Since VGG19 has good generalization ability and accuracy. Therefore, in PV resource assessment research, VGG19 can be used for feature extraction and classification of PV resource images to achieve the assessment of PV resources, improve the accuracy and efficiency of PV resource assessment, and provide a more reliable basis for the planning and design of PV power generation [37].

In VGG19, the input matrix is filtered by different convolutional kernels [38], features are extracted, and then the resulting activation mappings are stacked [39], and the features extracted by the convolutional layers are obtained after inputting the activation function. The output $x_{j}^{n}$ of the convolution operation in the convolution layer is:

(1) $$x_{j}^{n}=f\left(\sum_{i\in p_{j}}x_{i}^{n-1}k_{ij}^{n}+b_{j}^{n}\right)$$

where *f* ( ) is the activation function; $p_{j}$ is the position of the convolution kernel when processing the input; $x_{i}^{n-1}$ is the input corresponding to the *n*-1st layer on the *i-th* window; $k_{ij}^{n}$ is the weight matrix; $b_{j}^{n}$ is the bias matrix; $x_{j}^{n}$ is the output of the convolution operation of the convolution layer;

The pooling layer of VGG19 follows the convolutional layer, and the pooling layer moves through the input sequence in a sliding form and performs the computation, and the output $y_{j}^{n}$ of the pooling layer is:

(2) $$y_{j}^{n}=f\left(pool\left(y_{i}^{n-1}\right)+b_{j}^{n}\right)$$

where $y_{i}^{n-1}$ is the input corresponding to the *n*-1st layer in the pooling layer at the *i-th* window position; *f* ( ) is the activation function; $b_{j}^{n}$ is the bias matrix; $y_{j}^{n}$ is the output of the *n*-th layer after pooling; and the *pool* is the pooling function;

#### 3.1.3 An algorithmic framework based on Xception and VGG19 two-layer network

In this study, we propose an algorithmic framework based on Xception and the VGG19 two-layer network. In the study of PV resource assessment, a two-layer network framework combining the Xception algorithm and VGG19 algorithm is used in this study. The input of the framework is a PV image and the output is the PV resource prediction corresponding to that image. This two-layer network framework is a cascade combination of the Xception algorithm and the VGG19 algorithm to form a two-layer neural network structure. By cascading operations, the advantages of the powerful feature extraction capability of VGG19 and the powerful regression prediction capability of the Xception algorithm can be combined. The advantage of this combination is that the VGG19 network excels in extracting local features of the image, while the Xception network excels in extracting global features. With the two-layer network structure, the advantages of both networks can be fully utilized to improve the accuracy and stability of PV resource prediction. At the same time, the combined method can also avoid the overfitting problem caused by using a single algorithm and improve the generalization performance of the model, which has a high practical value.

**Fig. 3:**
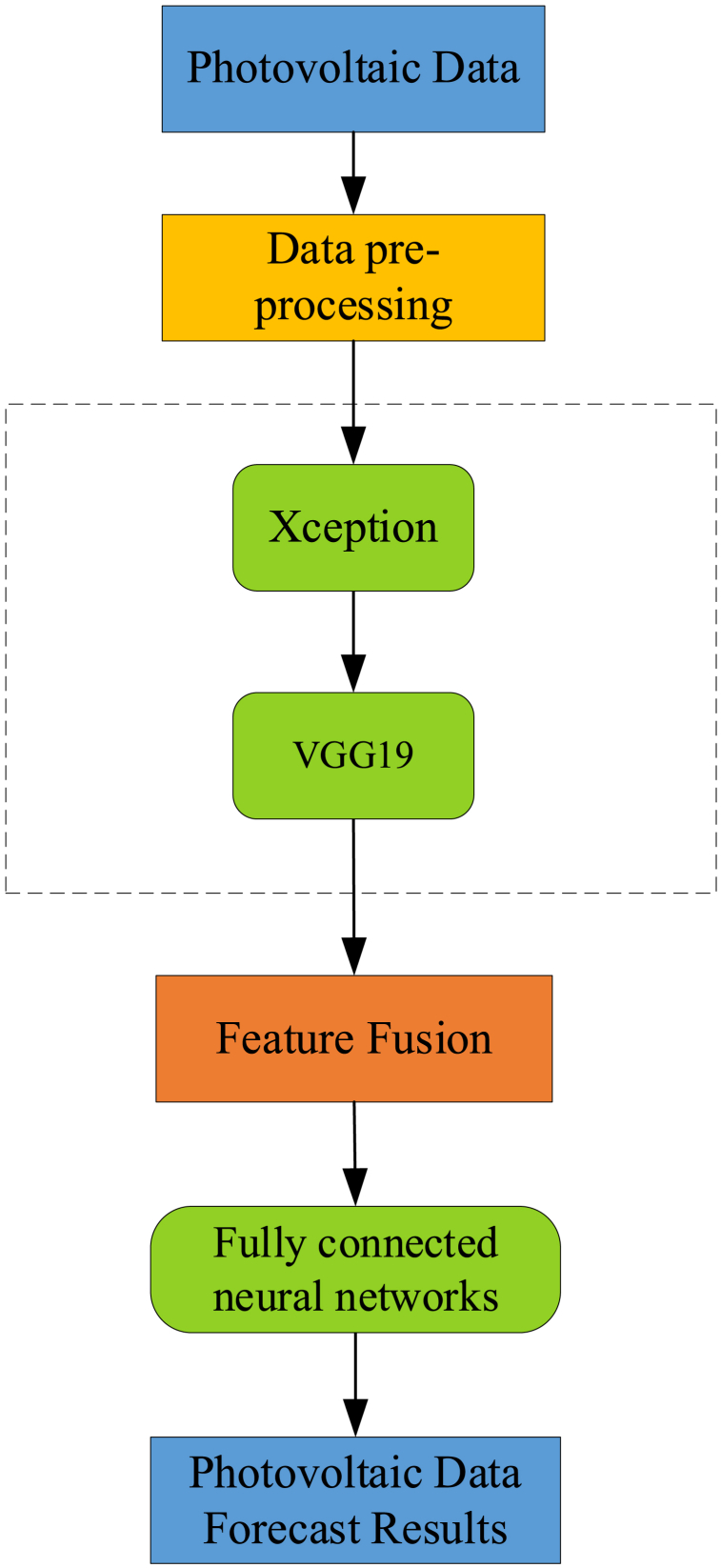
Framework diagram of PV resource assessment method based on Xception and VGG19 two-layer network algorithm.

The framework of the PV resource assessment method based on Xception and the VGG19 two-layer network algorithm is shown in Fig. 3. In the framework proposed in this paper, the PV data are first preprocessed and converted to image format. Then, the images are feature extracted using Xception and VGG19 two-layer networks. In this two-layer network structure, VGG19 is used as the first layer network, and the VGG19 algorithm performs feature extraction on PV images to obtain a more abstract feature representation. Xception is used as the second layer network, which performs deeper feature extraction based on the features extracted by VGG19, further improving the performance of the algorithm. Finally, the feature vectors extracted by the two feature extractors are feature fused to obtain a combined feature vector. The fused features are fed into a fully connected neural network [40] for regression prediction, and the final prediction results for PV data are obtained.

Therefore, the pseudo-code based on the Xception and VGG19 bilayer network algorithm proposed in this study is shown in Algorithm 1.

**Table:** 

Algorithm 1. Pseudocode based on Xception and VGG19 bilayer network algorithm
1: Input the solar irradiation intensity
2. Initialize parameters
3: Data pre-processing (normalization operation on imported PV data)
4: Constructing the Xception model 5: Construction of VGG19 model 6: Training Xception model and VGG19 model with training data and labels 7: The trained Xception model performs feature extraction on the input PV image according to the steps in Fig. 1 to obtain a more abstract feature representation
8: The trained VGG19 model performs deeper feature extraction based on the features extracted by the VGG19 model according to the steps in Fig. 2 and the formulas in Equations (1), (2) 9: The feature vectors extracted by the two feature extractors are feature fused to obtain a combined feature vector
10: Input the feature fusion data into a fully connected neural network for regression prediction
11: Output the results of solar radiation intensity prediction

Therefore, the features of the PV resource assessment method based on Xception and VGG19 two-layer network algorithm proposed in this study can be summarized as:

Because of the improved feature extraction effect of the Xception algorithm by separable convolutional layer structure, it is capable of capturing more fine-grained feature information. VGG19 uses a deeper convolutional neural network structure that is capable of extracting higher-level abstract features. Therefore, combining the two algorithms can make full use of the advantages of both algorithms to improve the feature expression in PV resource assessment.

This research forms a two-layer network framework by combining Xception with the VGG19 algorithm. The proposed two-layer network framework can avoid the limitations of a single algorithm and enhance the robustness of the model. In addition, the proposed two-layer network framework can provide a comprehensive and detailed assessment of PV resources by fusing multiple feature extraction methods, which improves the adaptability of the model to different scenarios and changing conditions.

Xception has better interpretability compared to traditional convolutional neural networks, and its network structure is easier to understand and interpret. By combining Xception and VGG19 algorithms, more detailed feature representation and interpretation can be provided, making the evaluation results of the model more interpretable.

### 3.2 Evaluation indexes

In the study of PV resource assessment, the evaluation indexes of regression prediction models can be used to evaluate and optimize the prediction models to obtain more accurate and stable PV resource prediction results. In this study, the following metrics are used to evaluate the models.

(1) Mean squared error: MSE [41] is used to measure the degree of difference between the predicted and true values of the model. The smaller the MSE (Equation (3)), the better the predictive ability of the model.

(3) $$\text{MSE}=\frac{1}{n}{\sum \left(y_{i}-\bar{y_{i}}\right)}^{2}$$

where *n* is the sample size; $y_{i}$ is the true value of PV data; $\bar{y_{i}}$ is the predicted value of PV data.

(2) root mean squared error: The RMSE [42] metric takes the square root of the mean of the sum of squares of the errors. The smaller the value of the RMSE (Equation (4)), the better the prediction of the model.

(4) $$\text{RMSE}=\sqrt{\frac{1}{n}\sum_{i=1}^{n}{\left(y_{i}-\bar{y_{i}}\right)}^{2}}$$

where *n* is the sample size; $y_{i}$ is the true value of PV data; $\bar{y_{i}}$ is the predicted value of PV data.

(3) Mean absolute error: The MAE [43] indicator seeks the mean of the absolute value of the error. The smaller the value of MAE (Equation (5)), the better the prediction of the model.

(5) $$\text{MAE}=\frac{1}{n}\sum_{i=1}^{n}\left|{\hat{y}}_{i}-\bar{y_{i}}\right|$$

where *n* is the number of samples; ${\hat{y}}_{i}$ is the true value of PV data; $\bar{y_{i}}$ is the predicted value of PV data.

(4) R^2^：R^2^ [44] indicates how much of the variation in the dependent variable can be explained by the independent variable. The larger R^2^ (Equation (6)) indicates the better explanatory power of the model.

(6) $$R^{2}=1-\frac{\text{SSR}}{\text{SST}}$$

where SSR is the sum of squared residuals; SST is the sum of total squares.

## 4 Cases studies

The hardware configuration of the computer used for the simulation experiments in this study is as follows: the system is Windows 11, the processor is Intel (R) Core (TM) i7-12900H, the main frequency is 2.30 GHz, and the memory is 16 GB. The simulation environment is MATLAB 2021b. To increase the control of simulation experiments, two groups of comparison algorithms are set up in this study, namely VGG19 and Xception algorithm.

### 4.1 Study area and data collection

**Fig. 4:**
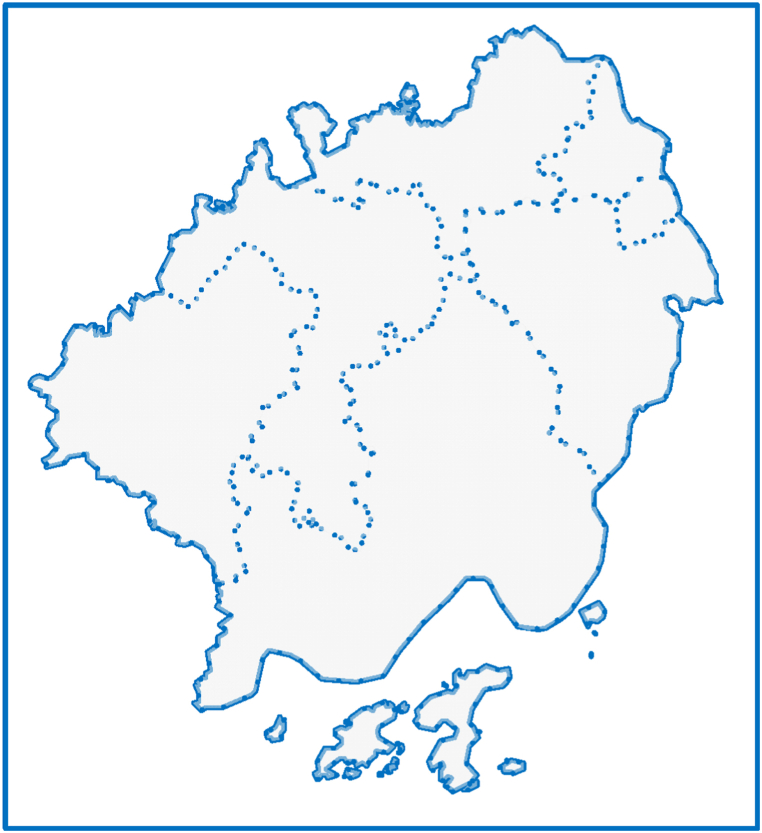
Jiangmen city location map.

The geographical location of Jiangmen City is shown in Fig. 4. The city is located in the southwestern part of Guangdong Province, China, at the northwestern end of the Pearl River Delta, adjacent to the mouth of the Pearl River, with geographical coordinates of 22°28′ to 22°51′ N latitude and 113°02′ to 113°13′ E longitude. The experimental data of this study were obtained from the daily site irradiation intensity from May 13, 2022, to November 11, 2023, measured by the China PV Test Network for Jiangmen Jiang Powder Magnet PV power plant in Guangdong.

### 4.2 Solar irradiation intensity prediction based on Xception algorithm

To obtain a better prediction performance of the Xception model, it is necessary to select suitable parameters for simulation experiments. In the Xception algorithm model, the learning rate of the Xception algorithm [45] is set to 0.01, the batch size [46] is set to 32, and the activation function of the Xception algorithm is the ReLU function [47] to obtain a faster convergence rate, higher model accuracy, and improved generalization capability of the model. Table 1 shows some of the parameter types in the Xception model and the values of each parameter type.

**Table 1:** Hyperparameters in the Xception model.

Hyperparameter Type	Value
Learning Rate	0.01
Batch size	32
Activation function	ReLU
Optimizer	Adam

**Fig. 5:**
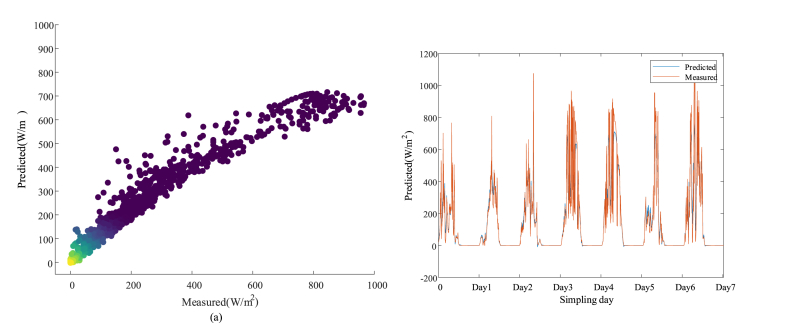
Plots of Xception model predicted solar radiation intensity results: (a) scatter plot of Xception model predicted solar radiation intensity; (b) line plot of Xception model predicted solar radiation intensity.

Fig. 5 shows the results of solar radiation intensity obtained based on the Xception model prediction. Fig. 5 (a) shows the scatter plot of the solar radiation prediction results obtained based on the Xception model. Meanwhile, to observe the good and bad solar radiation intensity prediction based on the Xception model more visually, Fig. 5 (b) shows the line graph of solar radiation intensity prediction results obtained based on the Xception model.

### 4.3 Solar irradiation intensity prediction based on VGG19 algorithm

To improve the prediction performance of the VGG19 model, accelerate the convergence speed, and effectively avoid the gradient disappearance problem [48], the VGG19 algorithm hyperparameters are shown in Table 2.

**Table 2:** Hyperparameters in the VGG19 model.

Hyperparameter Type	Value
Learning Rate	0.001
Convolutional layer	19
Activation function	ReLU
Optimizer	Adam

**Fig. 6:**
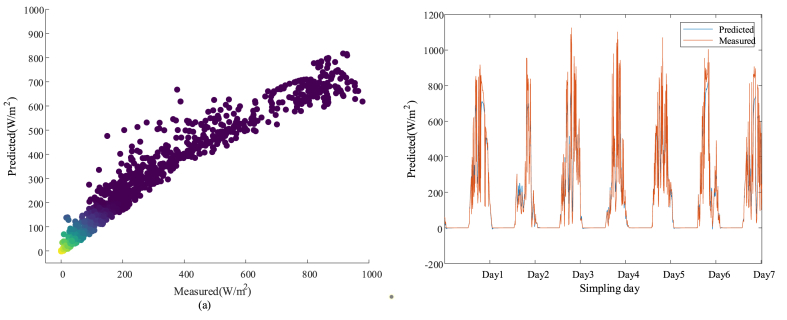
Plots of solar radiation intensity results predicted by the VGG19 model: (a) scatter plot of solar radiation intensity predicted by the VGG19 model; (b) line graph of solar radiation intensity predicted by the VGG19 model.

Fig. 6 shows the solar radiation intensity results obtained based on the VGG19 model prediction. Fig. 6 (a) shows the scatter plot of the solar radiation prediction results obtained based on the VGG19 model. Meanwhile, to observe more visually the good and bad solar radiation intensity prediction based on the VGG19 model, Fig. 6 (b) shows the line graph of solar radiation intensity prediction results obtained based on the VGG19 model.

### 4.4 Solar irradiation intensity prediction based on Xception and VGG19 two-layer network algorithm

The proposed method combines the Xception network and VGG19 convolutional neural network, combining the advantages of both algorithms to give full play to their respective strengths, which can improve the robustness of the model and the accuracy of the PV resource assessment.

Table 3 lists the parameters involved in the two comparison algorithms used in this study.

**Table 3:** Hyperparameters in VGG19, Xception.

Algorithm Type	Hyperparameter Type	Value
VGG19	Learning Rate	0.001
	Convolutional layer	19
	Activation function	ReLU
	Optimizer	Adam
Xception	Learning Rate	0.01
	Batch size	32
	Activation function	ReLU
	Optimizer	Adam
	Learning Rate	0.01
Inception V3	Activation function	ReLU
	Optimizer	Adam

**Fig. 7:**
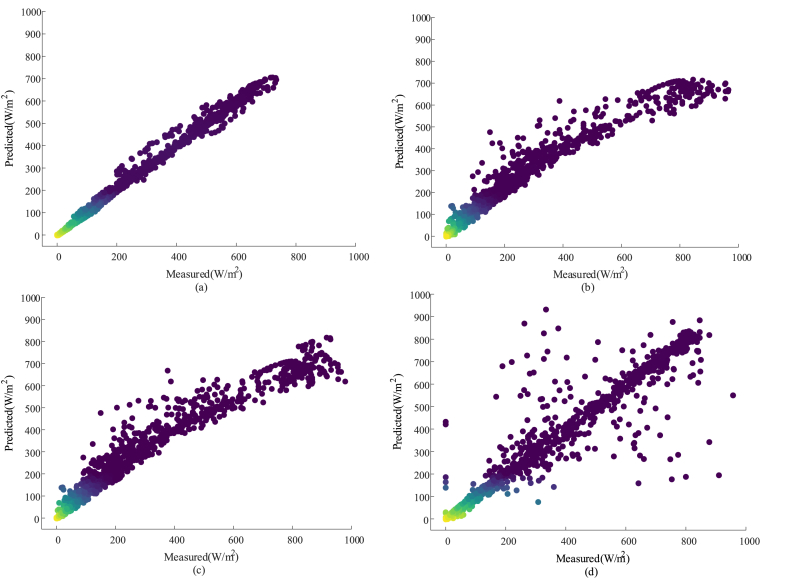
Scatterplots of solar radiation intensity results output by three algorithms: (a) scatterplot of solar radiation intensity predicted by two-layer network algorithm model; (b) scatterplot of solar radiation intensity predicted by Xception model; (c) scatterplot of solar radiation intensity predicted by VGG19 model; (d) scatterplot of solar radiation intensity predicted by Inception V3 model.

Fig. 7 (a) shows the scatter plot of the predicted solar radiation intensity obtained from the Xception and VGG19 two-layer network algorithm-based model proposed in this study. Fig. 7 (b) shows the scatter plot of the predicted solar radiation intensity obtained from the Xception-based algorithm model proposed in this study. Fig. 7 (c) shows the scatter plot of the predicted solar radiation intensity obtained from the VGG19 algorithm-based model proposed in this study. Fig. 7 (d) shows the scatter plot of the predicted solar radiation intensity obtained from the VGG19 algorithm-based model proposed in this study. As shown in Fig. (7), the performance of the two-layer network structure consisting of the VGG19 convolutional neural network and the Xception algorithm is significantly outperformed by the performance of the single structure of the VGG19 and Xception, and Inception V3 algorithms.

To scientifically evaluate the prediction performance of the proposed method and the comparison algorithms, the values of MSE, RMSE, MAE, and R^2^ evaluation metrics of the three algorithms were calculated in this study. The evaluation index values for each type of algorithm are shown in Table 4. As can be seen from Table 4, the values of MSE, RMSE, and MAE evaluation indexes calculated by the proposed two-layer network algorithm based on Xception and VGG19 in this study are smaller than those calculated by the VGG19 algorithm and Xception algorithm, which verifies the feasibility and effectiveness of the proposed two-layer network algorithm, improves the accuracy of PV resource evaluation, and has high predictive stability and robustness.

**Table 4:** Evaluation index values for each type of algorithm.

Evaluation Indicators	MSE	RMSE	MAE	R^2^
Xception	3224.2104	56.7821	24.7010	0.9360
VGG19	4107.3986	64.0890	29.7381	0.9365
Inception V3	4209.2465	69.6094	22.8935	0.9336
Proposed method	239.7568	15.4841	6.9073	0.9951

It can be inferred from Table 4 that when a single method (i.e., VGG19 or Xception or Inception V3) is used, there is a large error between the predicted and measured values of solar irradiation intensity. It is mainly because of the limitations of each of these two models in feature extraction of power load data, which cannot fully capture the complex features and patterns in the data. However, by introducing a two-layer network algorithm, the respective advantages of VGG19 and Xception are fully utilized to improve the prediction accuracy. Such a stacked approach effectively reduces the prediction error because the two models work together and mutually compensate for the shortcomings of a single approach, which highlights the advantages of the algorithm proposed in this study and makes it perform superior and more robust in solar irradiation intensity prediction.

## 5 Discussion

Compared to single network algorithms and frameworks, two-layer network algorithms and two-layer network frameworks can provide deeper feature extraction of the input data, resulting in higher prediction accuracy.

Although the Xception and VGG19 two-layer network-based algorithm proposed in this study has some advantages in PV resource assessment, there are still some shortcomings and drawbacks. There are a large number of hyperparameters in the two-layer network framework that need to be adjusted, including the learning rate, and weight decay. In this study, the learning rate in the Xception algorithm, the VGG19 algorithm, and the two-layer network algorithm proposed in this study need to be manually adjusted in a time-consuming manner to improve the prediction performance of the algorithm. Adjustment of manual parameters affects the length of training time and training results.

To address the problems of the method proposed in this study, optimization methods of algorithms can be explored to address the problems of high training complexity, difficult parameter tuning, and high model complexity.

## 6 Conclusions

To evaluate the PV resources more accurately, a two-layer network structure framework is proposed. A two-layer network algorithm is then proposed for the two-layer network structure framework. The feasibility and effectiveness of the two-layer network algorithm for the PV resources assessment problems are verified under case studies. The main research work of this paper is as follows.

(1) The two-layer network algorithm proposed in this study is utilized in the proposed two-layer network framework. The values of the evaluation metrics calculated by the two-layer network algorithm proposed in this study are all smaller than the other compared algorithms.
(2) The proposed two-layer network framework can capture the correlation between data features more accurately than a single network framework, improving the stability and reliability of the model prediction.
(3) Simulation results show that the MSE, RMSE, and MAE values obtained from the proposed two-layer network algorithm and two-layer network framework in this study are minimized. The method proposed in this study can predict the trend of PV resources more accurately.

## Data availability statement

Data associated with this study are not deposited in publicly available repositories. Data will be made available on request.

## CRediT authorship contribution statement

**Lifeng Li:** Methodology. **Zaimin Yang:** Software. **Xiongping Yang:** Data curation. **Jiaming Li:** Project administration. **Qianyufan Zhou:** Writing – review & editing.

## Declaration of competing interest

The authors declare that they have no known competing financial interests or personal relationships that could have appeared to influence the work reported in this paper.

## Appendix A

GA: Genetic algorithm
MSE: Mean squared error
MAE: Mean absolute error
PV: Photovoltaic
RNN: Recurrent neural networks
RMSE: Root mean squared error
SVM: Support vector machines
SSR: Sum of squared residuals
SST: Sum of total squares

## Appendix B

*f*(.): Activation function
$b_{j}^{n}$: Bias matrix
$x_{i}^{n-1}$: Input corresponding to the *n*-1st layer on the *i-th* window
$y_{i}^{n-1}$：: Input corresponding to the *n*-1st layer in the pooling layer at the *i-th* window position
$x_{j}^{n}$: Output of the convolution operation of the convolution layer
$y_{j}^{n}$：: Output of the *n*-th layer after pooling
$p_{j}$: Position of the convolution kernel
$\bar{y_{i}}$: Predicted value of PV data
*n*: Sample size
$y_{i}$: True value of PV data
$k_{ij}^{n}$: Weight matrix

6 of 20.

## References

[1] Dong F., Li Y., Gao Y. (2022). Energy transition and carbon neutrality: exploring the non-linear impact of renewable energy development on carbon emission efficiency in developed countries. Resour. Conserv. Recycl..

[2] Schindele S., Trommsdorff M., Schlaak A. (2020). Implementation of agrophotovoltaics: techno-economic analysis of the price-performance ratio and its policy implications. Appl. Energy.

[3] Zhang P., Li W., Li S. (2013). Reliability assessment of photovoltaic power systems: review of current status and future perspectives. Appl. Energy.

[4] Izam N.S.M.N., Itam Z., Sing W.L. (2022). Sustainable development perspectives of solar energy technologies with focus on solar Photovoltaic—a review. Energies.

[5] Carneiro T.C., de Carvalho P.C.M., Alves dos Santos H. (2022). Review on photovoltaic power and solar resource forecasting: current status and trends. J. Sol. Energy Eng..

[6] Ozcanli A.K., Yaprakdal F., Baysal M. (2020). Deep learning methods and applications for electrical power systems: a comprehensive review. Int. J. Energy Res..

[7] Alizamir M., Kim S., Kisi O. (2020). A comparative study of several machine learning based non-linear regression methods in estimating solar radiation: case studies of the USA and Turkey regions. Energy.

[8] Yin W., Kann K., Yu M. (2017).

[9] Kurani A., Doshi P., Vakharia A. (2023). A comprehensive comparative study of artificial neural network (ANN) and support vector machines (SVM) on stock forecasting. Annals of Data Science.

[10] Katoch S., Chauhan S.S., Kumar V. (2021). A review on genetic algorithm: past, present, and future. Multimed. Tool. Appl..

[11] Mirjalili S., Song Dong J., Sadiq A.S. (2020). Genetic algorithm: theory, literature review, and application in image reconstruction. Nature-Inspired Optimizers: Theories, Literature Reviews and Applications.

[12] Efficient Computation on Recurrent Neural Networks Using Compressed Models.

[13] Abualigah L., Diabat A. (2020). A comprehensive survey of the Grasshopper optimization algorithm: results, variants, and applications. Neural Comput. Appl..

[14] A Review on Data-Driven Approaches for Solar Photovoltaic Power Forecasting.

[15] Ağbulut Ü., Gürel A.E., Biçen Y. (2021). Prediction of daily global solar radiation using different machine learning algorithms: evaluation and comparison. Renew. Sustain. Energy Rev..

[16] Song C., Wang Q., Wang Y. (2021).

[17] Kassani S.H., Kassani P.H., Khazaeinezhad R. (2019).

[18] Mascarenhas S., Agarwal M. (2021). A comparison between VGG16, VGG19 and ResNet50 architecture frameworks for image classification[C]//2021 international conference on disruptive technologies for multi-disciplinary research and applications (CENTCON). IEEE.

[19] Liu E.Z., Haghgoo B., Chen A.S. (2021). Just train twice: improving group robustness without training group information[C]//International Conference on Machine Learning. PMLR.

[20] Deep Learning Ensembles for Time Series Forecasting: Recent Advances and Perspectives.

[21] Bashir D., Montañez G.D., Sehra S. (2020).

[22] Shan J., Lu S., Liu S. (2022).

[23] Wu G., Yu M., Shi W. (2020). Image recognition in online monitoring of power equipment. Int. J. Adv. Rob. Syst..

[24] Lucas Segarra E., Ramos Ruiz G., Fernández Bandera C. (2020). Probabilistic load forecasting for building energy models. Sensors.

[25] Chen B., Ju X., Xiao B. (2021). Locally GAN-generated face detection based on an improved Xception. Inf. Sci..

[26] Ahmed M.D.F., Mohanta J.C., Sanyal A. (2023). Path planning of unmanned aerial systems for visual inspection of power transmission lines and towers. IETE J. Res..

[27] Yermoldina G.T., Suimenbayev B.T., Sysoev V.K. (2019). Features of space solar power station control system. Acta Astronaut..

[28] Shi W., Zhu Y., Huang T. (2017). An integrated data preprocessing framework based on Apache spark for fault diagnosis of power grid equipment. Journal of Signal Processing Systems.

[29] Zhu K., Deng B., Zhang P. (2020). System efficiency and power: the bridge between the device and system of a thermoelectric power generator. Energy Environ. Sci..

[30] Divina F., Goméz Vela F.A., García Torres M. (2019). Biclustering of smart building electric energy consumption data. Appl. Sci..

[31] Jobayer M., Shaikat M.A.H., Rashid M.N. (2023).

[32] Son Y., Zhang X., Yoon Y. (2023). LSTM–GAN based cloud movement prediction in satellite images for PV forecast. J. Ambient Intell. Hum. Comput..

[33] Dai Q., Huo X., Hao Y. (2023). Spatio-temporal prediction for distributed PV generation system based on deep learning neural network model. Front. Energy Res..

[34] Zhao Y., Zhang H., Hu X. (2022). Penalizing gradient norm for efficiently improving generalization in deep learning[C]//International Conference on Machine Learning. PMLR.

[35] Deep Convolutional Neural Networks for Image Classification: A Comprehensive Review. 10.1162/NECO_a_00990 28599112

[36] Ball J.E., Anderson D.T., Chan C.S. (2017). Comprehensive survey of deep learning in remote sensing: theories, tools, and challenges for the community. J. Appl. Remote Sens..

[37] Scherer D., Müller A., Behnke S. (2010).

[38] Pavlović T.M., Radonjić I.S., Milosavljević D.D. (2012). A review of concentrating solar power plants in the world and their potential use in Serbia. Renew. Sustain. Energy Rev..

[39] Wang Z., Yang Y., Liu Z. (2023).

[40] Rajinikanth V., Joseph Raj A.N., Thanaraj K.P. (2020). A customized VGG19 network with concatenation of deep and handcrafted features for brain tumor detection. Appl. Sci..

[41] Shi D., Zhou J., Wang D. (2022). Research status, hotspots, and evolutionary trends of intelligent education from the perspective of knowledge graph. Sustainability.

[42] Chicco D., Warrens M.J., Jurman G. (2021). The coefficient of determination R-squared is more informative than SMAPE, MAE, MAPE, MSE and RMSE in regression analysis evaluation. PeerJ Computer Science.

[43] Ćalasan M., Aleem S.H.E.A., Zobaa A.F. (2020). On the root mean square error (RMSE) calculation for parameter estimation of photovoltaic models: a novel exact analytical solution based on Lambert W function. Energy Convers. Manag..

[44] Arunrat N., Pumijumnong N., Sereenonchai S. (2020). Factors controlling soil organic carbon sequestration of highland agricultural areas in the mae chaem basin, northern Thailand. Agronomy.

[45] Khayami S., Ekhlassi A., Rahbar M. (2023). Effect of earth-sheltering and atrium form and proportion integration on energy and lighting performance optimization in a hot arid climate of Mashhad, Iran. Energy Efficiency.

[46] Huang F., Enhanced adaptive gradient algorithms for nonconvex-PL minimax optimization, arXiv preprint arXiv:2303.03984 (2023). 10.48550/arXiv.2303.03984.

[47] Zhao Y., Zhang H., Hu X. (2022). Penalizing gradient norm for efficiently improving generalization in deep learning[C]//International Conference on Machine Learning. PMLR.

[48] Timor N., Vardi G., Shamir O. (2023). Implicit regularization towards rank minimization in relu networks[C]//International Conference on Algorithmic Learning Theory. PMLR.

